# The Novel Association of a Single Nucleotide Variant in the *COL3A1* Gene with Diffuse Coronary Aneurysms

**DOI:** 10.3390/cimb47020082

**Published:** 2025-01-27

**Authors:** Charlene Norgan Radler, Kevin Ku, Alison Hodge, Tianci Wang, Peyton Moore, Mohanakrishnan Sathyamoorthy

**Affiliations:** 1 Sathyamoorthy Laboratory, Department of Medicine, Burnett School of Medicine at TCU, Fort Worth, TX 76104, USAt.wang17@tcu.edu (T.W.);; 2 Consultants in Cardiovascular Medicine and Science, Fort Worth, TX 76104, USA; 3 Fort Worth Institute for Molecular Medicine and Genomics Research, Fort Worth, TX 76104, USA

**Keywords:** *COL3A1*, coronary artery aneurysm, coronary artery ectasia, single nucleotide variant, collagen matrix protein, extracellular matrix

## Abstract

The *COL3A1* gene, encoding the pro-alpha chain of type III collagen, has been implicated in a range of collagen-mediated diseases such as Ehlers–Danlos syndrome and aortic aneurysms. In this report, we present evidence for the first time associating a single nucleotide variant p.P517R in exon 22 of *COL3A1* with the development of diffuse coronary aneurysms in a human subject without prior atherosclerotic cardiovascular disease, connective tissue disorder, or phenotypic characteristics diagnostic for vascular Ehlers–Danlos syndrome. Computational modeling of this specific variant in AlphaFold and in silico analyses predict deleterious alterations in the structure and function of the *COL3A1* gene product, alpha 1 chain of type III collagen. This novel phenotype-to-genotype correlation should prompt further investigation into the mechanistic basis of this association.

## 1. Introduction

Coronary artery aneurysms (CAA) are characterized by the localized dilatation of an epicardial coronary artery exceeding the diameter of adjacent normal segments by 50% or more. In contrast, coronary artery ectasias (CAE) are distinguished by their diffuse extension over more than one-third of the coronary artery length [1,2,3]. Coronary artery aneurysms and ectasias involve all three layers of the tunica intima, media, and adventitia [4]. Although their pathogenesis is not fully understood, atherosclerosis is known to be the most common cause via a mechanism of hyalinization and lipid deposition in the vessel tunica intima and media, leading to vessel wall weakening. Symptoms can range from clinically silent, incidental findings on angiogram or computed tomography to the spectrum of acute coronary syndrome, acute cardiac tamponade, or sudden death [1,4].

While advances in diagnostics have yielded a basic epidemiology of CAA via retrospective analysis of large registries, limitations include observational design and variations in patient inclusion criteria and angiographic criteria [4]. CAA has an estimated incidence ranging from 0.35% to 4.9%, as reported by studies including the international Coronary Artery Aneurysm Registry (CAAR) and the Coronary Artery Surgery Study (CASS) [5,6,7]. The incidence of CAA appears to vary regionally, suggesting influence from genetic and environmental factors. In one study using national health data, the incidence rate of CAA and CAE on angiography in Taiwan was found to be 0.87 per 10^5^ person-years from 2005 to 2011 [8]. Another study performed at a hospital in Shanghai, China, found the overall incidence of CAA and CAE on angiography to be 1.92% from 2014 to 2022 [9].

CAAs are most commonly develop in the left anterior descending artery (49.6%), followed by the right coronary artery (31.4%) and the circumflex artery (27.5%), according to recently published data from the CAAR international registry [10]. While CAAs derived from atherosclerotic and vasculitis etiologies often affect more than one artery, the involvement of three coronary vessels or the left main is rare [1,11]. Congenital and iatrogenic CAAs typically affect a single vessel.

While the pathogenesis of CAA and CAE remains under investigation, the underlying mechanisms appear to contribute to the weakening and dilation of the vessel wall [4]. Known etiologies include atherosclerotic disease, vasculitis disorders such as Kawasaki disease and Takayasu arteritis, hereditary connective tissue disorders including Marfan syndrome and Ehlers–Danlos syndrome, infection, iatrogenic injury, and congenital disease. Current data on the long-term outcomes of a large, multicenter registry of adult patients with CAAs demonstrated a risk factor profile for atherosclerotic cardiovascular disease (ASCVD), with 85.6% of the cohort demonstrating concomitant severe coronary artery disease (CAD) [10]. While the prevalence of connective tissue disorders among the cohort was low (2%; 35/1729), the exclusion of patients with isolated CAE from the registry may similarly exclude etiologies leading to a more severe aneurysmal phenotype.

The vascular type of Ehlers–Danlos syndrome (vEDS) is caused by mutations in *COL3A1*, which encodes the pro-alpha 1 chain of type III collagen [12]. This severe form of EDS is characterized by the spontaneous rupture of large arteries and other hollow organs, which can result in sudden death. *COL3A1* mutations are additionally associated with arterial aneurysms and fibrotic disease. A large Dutch cohort study of 142 individuals with pathogenic or likely pathogenic *COL3A1* variants identified five patients with spontaneous coronary artery dissections at a mean age of 39 years [13]. Notably, one of the five patients lacked other phenotypic characteristics highly suggestive of vEDS. To the best of our knowledge, although few cases have been reported demonstrating spontaneous coronary artery dissection in patients with *COL3A1* variants and without a diagnosis of vEDS, none have reported coronary artery aneurysm or ectasia formation [14]. Although each formation is similar in pathophysiology, this report serves as a novel finding of coronary artery aneurysms and ectasias associated with a *COL3A1* variant in a patient who does not meet clinical criteria for vEDS.

Furthermore, there is limited work that describes any genetic associations between genes crucial to the extracellular matrix and coronary aneurysms. Genetic variants in *COL3A1* are most associated with aortic aneurysms secondary to the disruption of collagen integrity in the adventitia and media of the aorta, as demonstrated by a murine model [15]. Given the similarities in the components of the aorta and coronary vasculature, we hypothesize that pathogenic variants in *COL3A1* play a role in the development of CAAs and CAEs in a subset of patients. This report aims to advance the literature associating genetic variants with CAA and CAE development and prompt further mechanistic investigation into pathogenesis.

## 2. Clinical Features

**Figure 1:**
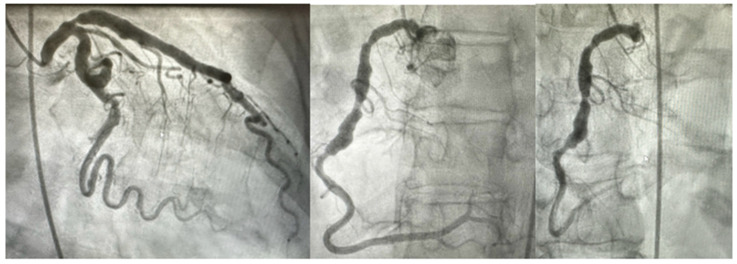
(**Left** Panel): Angiography of the left coronary circulation with aneurysm. (**Middle** and **Right** Panels): Angiography of the Right coronary circulation demonstrating diffuse coronary aneurysms.

A 67-year-old Caucasian female with risk factors for atherosclerotic vascular disease (ASCVD), including hyperlipidemia (well controlled on therapy), hypertension, and a family history of CAD, suffered an inferior ST-elevation myocardial infarction (STEMI) and underwent primary percutaneous coronary intervention (PCI) of the right coronary artery with a fifth-generation drug-eluting stent (RCA) at an outside institution. She sought care in our program and underwent a thorough examination, which yielded no overt joint hypermobility, loose skin, or any auscultation abnormalities. Standard biochemical laboratories and more advanced parameters, such as a Lipoprotein A level, were within standard reference ranges. A review of her coronary angiography demonstrated significant coronary aneurysms in all vessels (Figure 1). The patient’s post-hospitalization echocardiogram revealed a left ventricle of normal size with an ejection fraction of 60–65% qualitatively and 62% by Teicholz without any significant valvular findings, normal intracardiac pressures, and normal post-infarct subsegmental wall motion and contractility. By ultrasound, the ascending aorta measured 32 mm with a normal measurement of the arch and had a normal Doppler evaluation. Her ostial LAD stenosis (Figure 1) was managed with a step-wise intensification of medical therapy. After three months of dual antiplatelet therapy, she underwent a successful and uncomplicated single vessel left internal mammary artery (LIMA) to left anterior descending (LAD) procedure. Given the diffuse coronary ectasia/aneurysms, she was offered clinical genetic testing to guide longitudinal clinical care, including surveillance plans for arterial aneurysms elsewhere. Over the patient’s time of care with us, by ultrasound assessment, her ascending aorta, aortic arch, proximal descending aorta, and carotid/vertebral arteries have had normal pulsatile flow and normal morphology without aneurysmal changes. Further CT angiographic screening is planned.

## 3. Materials and Methods

### 3.1. Next-Generation Sequencing Analysis

For genetic testing, we utilized a candidate gene approach with a panel of 35 genes associated with thoracic aneurysmal and dissection diseases (TAAD) (Table 1) using a standard commercially available genomic DNA saliva isolation kit comprising standard lysis/binding solutions, gDNA binding beads, and washing/elution solutions and run in a 96-well format [16]. The commercially available panel of 35 genes we chose represents a broad consensus of genes encoding endothelial, smooth muscle, and extracellular matrix components of the entire vascular wall, along with regulatory signaling genes such as the TGFB superfamily; this panel was updated from that originally reported in 2003 but was updated in 2023 to remain current to the literature [17]. Genetic sequencing was performed by Next-Generation or Sanger sequencing of all coding domains and well into the flanking 5′ and 3′ ends of all the introns and untranslated regions. Gross deletion/duplication analysis determines the gene copy number for the covered exons and untranslated regions of all genes (excluding CBS and TNXB exons 32–44). Bait-capture methods were utilized to enrich the coding exon sequences of interest using biotinylated oligonucleotide probes and subsequent polymerase chain reaction and sequencing, utilizing NCBI reference sequences (Table 1). Additional Sanger sequencing is performed for any regions missing or with any insufficient read depth coverage for reliable heterozygous variant detection. Variants in regions complicated by pseudogene interference, variant calls not satisfying depth of coverage, and potentially homozygous variants are verified by Sanger sequencing. Gross deletion/duplication analyses are performed for all genes using a custom pipeline based on read-depth from NGS data followed by a confirmatory orthogonal method, as needed. Sequence analysis of the above genes is based on the NCBI reference sequences, as listed in Table 1 [16]. The patient was given personalized genetic counseling in our practice.

### 3.2. Single Nucleotide Variant Analysis

Data were gathered from the Uniprot database regarding *COL3A1* function and domains [18]. The ClinVar database was accessed to investigate and compile data on the observed single nucleotide variant (SNV) [19]. The observed frequency of this variant among genetic ancestry groups was obtained from the Genome Aggregation Database (gnomAD) [20]. The properties of the original and substituted amino acid resulting from the SNV were compared, including the calculation of the Grantham Score [21,22].

The mutant structure was predicted and modeled in AlphaFold 3 using the *COL3A1* FASTA sequence with arginine substituted for proline at amino acid position 517 [23,24]. The top-ranked structure (0.7) was selected out of five predictions. Finally, in silico analysis was performed using the rare exome variant ensemble learner (REVEL) score, which combines scores from 13 individual tools, including PolyPhen-2 and MutPred [25]. REVEL has demonstrated high performance in distinguishing pathogenic variants from rare neutral variants with allele frequencies less than 0.5%.

## 4. Results

### Genetic Testing

**Figure 2:**
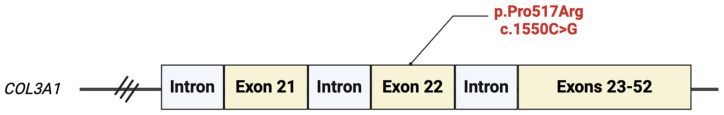
Simplified structure of the *COL3A1* gene demonstrating the isolated mutation in our patient, a proline to arginine substitution on exon 22. This results in the replacement of a non-polar amino acid with a basic amino acid, potentially responsible for the deleterious effect of the variant on the coronary arteries [26].

Genetic testing revealed only one variant of unknown significance (VUS) among this entire panel of 35 genes located in the *COL3A1* gene. The p.P517R variant, which is also known as the c.1550C>G variant, impacts the *COL3A1* gene in exon 22 (Figure 2) [18].

This p.P517R single nucleotide variant (SNV) has been reported five times in the ClinVar database for varying ECM pathologies, see Table 2 [19]. This variant has been identified in 56/1,565,758 chromosomes in the general population (0.003577%) by gnomAD, suggesting that this is not a common variation in the population [20]. The variant is only demonstrated in the European (non-Finnish) group in gnomAD and has a higher frequency among genetic females.

The p.P517R SNV results in a missense variant that substitutes a non-polar, ringed proline amino acid for a basic arginine amino acid at the 517 position, two amino acids with different properties (Table 3). The Grantham Score, a calculation used to predict the effect of amino acid substitutions, was 103 [21]. This elevated score is secondary to the varying properties of the two amino acids and suggests further evolutionary distance.

**Figure 3:**
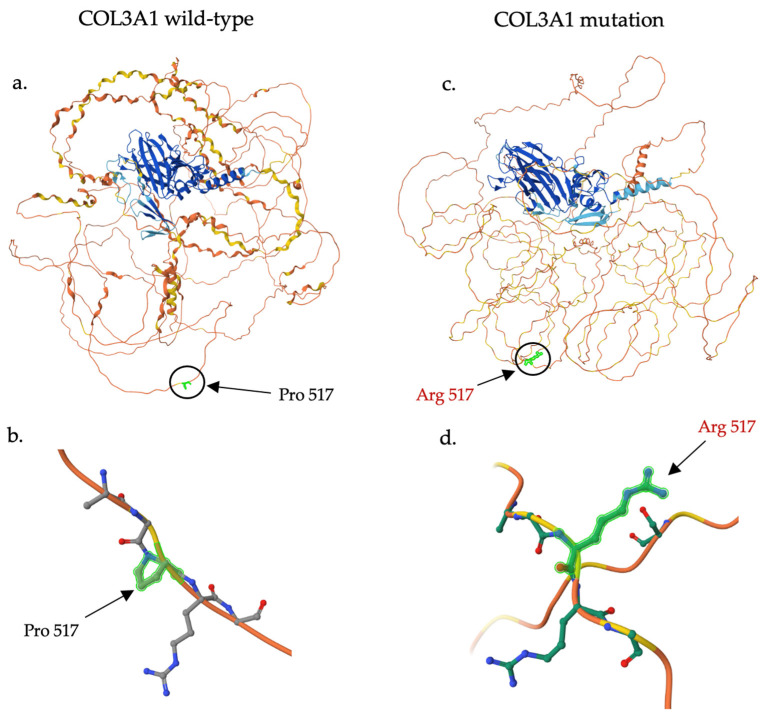
Three dimensional illustrative representations of *COL3A1* with p.P517R, predicted by AlphaFold [23,24]. Model color corresponds to per-residue confidence with very high confidence in dark blue (pLDDT > 90); confident in light blue (90 > pLDDT > 70); low confidence in yellow (70 > pLDDT > 50); and very low confidence in orange (pLDDT < 50). (**a**) Structure prediction of the wild-type *COL3A1* protein with proline circled at position 517 in lime green. (**b**) Focus on the wild-type proline at position 517 in lime green with surrounding residues within 5 Å. Oxygen atoms are colored as red, nitrogen atoms as blue, and carbon as gray. (**c**) Structure prediction of the mutated *COL3A1* protein (p.P517R) with arginine circled at position 517 in lime green. (**d**) Focus on the variant arginine at position 517 in lime green with surrounding residues within 5 Å. Oxygen atoms are colored as red, nitrogen atoms as blue, and carbon as green. AlphaFold structure predictions are freely available for both academic and commercial use under Creative Commons Attribution 4.0 (CC-BY 4.0) license terms.

In silico prediction suggests this variant may have a deleterious impact on protein structure and function, as demonstrated by a REVEL score of 0.717 [25]. Protein structure prediction analysis conducted in AlphaFold demonstrated the possible effect of the variant amino acid residue (p.P517R), see Figure 3 [23,24].

## 5. Discussion

We report for the first time the association of diffuse coronary artery ectasia and aneurysms with a single nucleotide variant of currently classified unknown significance (VUS), p.P517R within exon 22 of the *COL3A1* (collagen type III alpha 1 chain) gene. It is crucial to note that diffuse coronary artery aneurysms (CAA) and coronary artery ectasia (CAE) were present in all coronary vessels, including the left main coronary artery (Figure 1), which is a rare presentation of coronary aneurysmal dilation only occurring in an estimated 0.1% of the population [27]. There is increasing clinical interest in viewing newly identified variants as potential “biomarkers” for the development of these diseases as pathogenicity is established through further study. There is currently a lack of data regarding the genetic frequency and impact of variants on the structure and function of coronary arteries in the context of CAA and CAE development. Placing an emphasis on understanding how genetic variants can impact the integrity of coronary vasculature, similar to our programmatic efforts to characterize the effect of genetic variants on aortic integrity, will enhance screening methods for CAAs and CAEs prior to their development.

The *COL3A1* gene encodes the pro-alpha 1 chain of type III collagen, which is widely distributed in hollow organs such as blood vessels, gallbladder, bladder, and the uterus [12,18]. Type III collagen plays a crucial role in providing tensile strength to the extensible connective tissues within these organs. This property results from its intricate synthesis process involving the formation of a tri-helix from three pro-alpha 1 chains and further assembly of multiple tri-helices into a collagen fiber, stabilized by extensive enzyme-mediated crosslinks. Previous molecular investigations in mice showed that heterozygous *COL3A1* knockout or deletions compromised vascular integrity, resulting in aortic aneurysms, dissections, or decreased aorta strength due to decreased collagen levels or abnormal collagen structure, while homozygous deletions led to earlier mortality [15,28,29].

Vascular Ehlers–Danlos syndrome (vEDS) is a severe form of EDS caused by heterozygous pathogenic variants in *COL3A1*, with a common phenotypic presentation of cigarette paper-like scarring, translucent skin, easy bruising over bony protuberances, and laxity of finger joints [12]. Notably, our patient did not present with these characteristics or meet minimal criteria suggestive of the disorder, namely a family history of vEDS, arterial rupture or dissection at less than 40 years of age, unexplained sigmoid colon rupture, or spontaneous pneumothorax in the presence of other features consistent with vEDS [30]. Although spontaneous coronary artery dissections have been reported in patients with *COL3A1* variants and without a diagnosis of vEDS, this article presents a novel case of coronary artery aneurysms and ectasias associated with a *COL3A1* variant.

**Figure 4:**
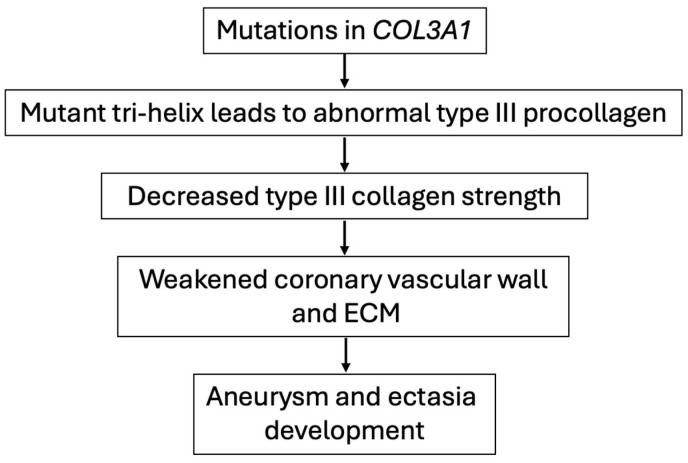
Proposed pathogenesis of *COL3A1* mutation observed in the subject leading to diffuse coronary artery aneurysms and ectasia. Adapted from Kuivaniemi and Tromp (2019) [12]. ECM = extracellular matrix.

Most research on *COL3A1* genetic variants in humans has focused on associations with Ehlers–Danlos syndrome, but several case studies have reported on patients with pathogenic mutations who are susceptible to aortic or arterial dissections, aneurysms, and ruptures [12,13,14,31,32,33]. In a recent review, Kuivaniemi et al. provided a comprehensive overview of *COL3A1*-associated diseases and possible pathogenic mechanisms [12]. These mechanisms include unstable mRNA products leading to nonsense-mediated mRNA decay (“null mutation”), increased degradation of the mutant polypeptide via the proteasomal system, and compromised collagen strength due to the incorporation of mutant polypeptide chains. Based on these mechanisms, the hypothesized pathogenic process of *COL3A1* mutations leading to the specific phenotype of diffuse coronary artery aneurysms and ectasia is illustrated in Figure 4.

Our patient’s mutation, p.P517R, is located in exon 22, within the triple helical region of the gene. A recent study by Pepin et al. analyzed clinical outcomes in 1231 individuals with heterozygous mutations across the length of the *COL3A1* gene [34]. The study found that single-nucleotide substitutions within the triple helical region, particularly glycine substitutions, account for over 60% of pathogenic mutations identified and were associated with the shortest average lifespan of 51 years [34]. This finding highlights the importance of the triple helical region in collagen fiber structure and integrity. Other locations of mutations occurring at high frequencies include those at RNA splice donor or acceptor sites and at the C-terminus due to its role in mediating the trimeric assembly of the procollagen chains [34,35,36].

Additionally, it is interesting to note that this patient’s mutation involves the substitution of a proline residue. The triple-helical domain is characterized by a repeated Gly-X-Y sequence contributing to the formation of the helical configuration. Proline and its derivative, hydroxyproline, are expressed in abundance within this region, frequently occupying the X or Y position in the Gly-X-Y repeat and helping stabilize the collagen chain via hydrogen bonds with glycine [37,38]. Although the impact of proline substitution has been studied less than that of glycine, our in silico analysis indicates a high likelihood that the variant is pathogenic, supporting the notion that this SNV has a deleterious impact on protein structure and function. Furthermore, the predicted mutant protein structure demonstrated a change in orientation between the substituted arginine at position 517 and its neighboring arginine at position 518, likely to minimize steric interactions between the two positively charged side chains. While further modeling is needed to predict the effect of this arginine-to-arginine interaction within the triple helix conformation, we hypothesize that this interaction may impact protein stability.

The aorta and coronary arteries have mostly analogous structures due to their similar functions as high-pressure vasculature. The walls of both include a tunica intima, media, and adventitia. The intima consists mostly of endothelial cells over a basement membrane, with laminin and collagen IV making up most of the layer. The media predominantly consists of vascular smooth muscle cells, elastin, and collagen I and III. The adventitia of the aorta and coronary arteries contains a thick connective tissue layer of collagen III studded with glycoproteins, fibroblasts, and inflammatory cells [39]. Due to the similar architecture of the vessels, we hypothesize that *COL3A1* mutations are associated with coronary artery aneurysmal disease, similar to their already-known role in the development of aortic aneurysmal disease. As of this report, no studies have examined the biophysical and biomechanical effects of specific *COL3A1* mutations on the development of CAAs and CAEs. Using data collected from genetic screening of patients with CAAs and CAEs, we would anticipate the development of animal transgenic models to probe the mechanistic relationship further.

## 6. Conclusions

CAAs and CAEs are localized dilations in the coronary arteries that may present as fatal acute coronary syndrome. Current literature regarding the genetic basis for CAA and CAE development is lacking. To the best of our knowledge, this report serves as the first to make an association between a variant in the *COL3A1* gene, which encodes the structural component of type III collagen, and the development of coronary artery aneurysms. Our hypothesis is further strengthened by showing that the identified variant is predicted to have deleterious effects on protein structure through in silico analysis. It is well-accepted in genetic medicine that single nucleotide variants may directly influence pathology alterations in encoded proteins or through epigenetic mechanisms that may influence the observed genotype-to-phenotype relationship. Further research is needed to elucidate the specific mechanisms of how mutations in the *COL3A1* gene lead to the development of CAAs and CAEs, which will ultimately advance screening and care for patients with this disease state.

## 7. Limitations

This report presents a novel and compelling association between COL3A1 and coronary aneurysms. However, we accept that this is in a single subject and should be confirmed through study in larger CAA cohorts. We also foresee the characterization of how this variant in *COL3A1* alters the collagen matrix in vivo, establishing a mechanistic basis between genetic alterations and effects at the cellular and tissue level.

## Figures and Tables

**Table 1:** The 35-gene panel and respective NCBI sequences utilized for genetic sequencing of the subject [16].

Gene	Sequence	Gene	Sequence	Gene	Sequence
*ACTA2*	NM_001613.2	*FKBP14*	NM_017946.2	*PRKG1*	NM_006258.3
*BGN*	NM_001711.4	*FLNA*	NM_001456.3	*SKI*	NM_003036.3
*CBS*	NM_000071.2	*FOXE3*	NM_012186.2	*SLC2A10*	NM_030777.3
*CHST14*	NM_130468.3	*LOX*	NM_002317.5	*SMAD3*	NM_005902.3
*COL1A1*	NM_000088.3	*MAT2A*	NM_005911.5	*SMAD4*	NM_005359.5
*COL1A2*	NM_000089.3	*MED12*	NM_005120.2	*TGFB2*	NM_003238.3
*COL3A1*	NM_000090.3	*MFAP5*	NM_003480.2	*TGFB3*	NM_003239.2
*COL5A1*	NM_000093.4	*MYH11*	NM_002474.2	*TGFBR1*	NM_004612.2
*COL5A2*	NM_000393.3	*MYLK*	NM_053025.3	*TGFBR2*	NM_003242.5
*EFEMP2*	NM_016938.4	*NOTCH1*	NM_017617.3	*TNXB* *	NM_019105.6
*FBN1*	NM_000138.4	*PLOD1*	NM_000302.3	*ZNF469*	NM_001127464.1
*FBN2*	NM_001999.3	*PRDM5*	NM_018699.2		

*: excludes exons 32–44.

**Table 2:** Reported phenotypes associated with the observed nucleotide variant [19].

Condition	Classification	Submissions
Ehlers–Danlos syndrome, type 4	Uncertain significance	2
Familial thoracic aortic aneurysm and aortic dissection	Uncertain significance	2
Not provided	Uncertain significance	1

**Table 3:** Comparison of amino acid properties present in p.P517R variant [22].

Properties	Proline	Arginine
Structure	Cyclic	Linear
Role	Introduces kinks in protein structures	Involved in hydrogen bonding and ionic interactions
Polarity	Hydrophobic	Hydrophilic
Side Chain pKa	Non-ionizable	~12.5 (highly basic)
Molecular Weight	115.13 g/mol	174.20 g/mol

## Data Availability

The data presented in this study are available on request from the corresponding author. The data are not publicly available due to patient privacy considerations.
